# Microbial Community Structure in the Taklimakan Desert: The Importance of Nutrient Levels in Medium and Culture Methods

**DOI:** 10.3390/biology13100797

**Published:** 2024-10-06

**Authors:** Feng Wen, Siyuan Wu, Xiaoxia Luo, Linquan Bai, Zhanfeng Xia

**Affiliations:** 1 Key Laboratory of Protection and Utilization of Biological Resources in Tarim Basin, College of Life Sciences and Technology, Xinjiang Production & Construction Corps, Tarim University, Alar 843300, China; fengwen0306@163.com (F.W.); wusiyuan_2022@163.com (S.W.); xxluo415@163.com (X.L.); 2 State Key Laboratory of Microbial Metabolism, Shanghai-Islamabad-Belgrade Joint Innovation Center on Antibacterial Resistances, School of Life Sciences and Biotechnology, Shanghai Jiao Tong University, Shanghai 200240, China; bailq@sjtu.edu.cn

**Keywords:** Taklimakan Desert, medium nutrient levels, oligotrophic, extreme environments, culture microorganisms

## Abstract

**Simple Summary:**

The Taklimakan Desert is a typical extreme environment of high temperature, drought, and barren soil. The microorganisms in this area have undergone long-term environmental adaptation, and the species distribution exhibits very regional characteristics. The traditional methods of isolation and culture cannot reflect the community composition of soil microorganisms completely. We used a simple yet effective strategy involving oligotrophic media, velvet cloth replicate, and extended culture times to not only reveal the distribution of bacteria in the Taklimakan Desert but also isolate a variety of rare and uncultured microorganisms and reveal the impact of nutrient levels on microbial diversity and culturability. Our findings highlight the utility of low-nutrient media in cultivating microbes from extreme environments, providing new perspectives into microbial diversity in desert ecosystems. We present a novel method for isolating microorganisms in nutrient-poor environments. This contributes to a deeper understanding of microbial diversity in extreme environments and offers new perspectives on how to effectively cultivate and study such microorganisms.

**Abstract:**

Although the Taklimakan Desert lacks the necessary nutrients and conditions to support an extensive ecosystem, it is a treasure trove of extremophile resources with special structures and functions. We analyzed the bacterial communities using oligotrophic medium and velvet cloth replicate combined with an extended culture duration. We isolated numerous uncultured microorganisms and rare microorganisms belonging to genera not often isolated or recently described, such as *Aliihoeflea*, *Halodurantibacterium*, and *Indioceanicola*. A total of 669 strains were isolated from the soil of the Taklimakan Desert, which were classified into 5 phyla, 7 classes, 25 orders, 42 families, 83 genera, and 379 species. Among them, 148 strains were potential new species. Our data show that even when working with samples from extreme environments, simple approaches are still useful for cultivating stubborn microbes. Through comparing the isolation effects of different nutrient levels on microbial diversity and abundance, the results show that reducing the nutrient level of the medium was more conducive to improving the culturability of microorganisms in low-nutrient environments, while the high-nutrient medium was more suitable for the isolation of dominant fast-growing strains. This study helps to better reflect the diversity of microbial resources and lays a foundation for the further research and utilization of soil microbial resources in the Taklimakan Desert.

## 1. Introduction

Microorganisms are undoubtedly the most abundant and diverse forms of cellular life on Earth and because of genomic advancements, culture is no longer necessary to obtain information about uncultured microorganisms. However, without laboratory cultures, predicted microbial growth, metabolism, and physiological properties cannot be defined, leaving numerous unanswered questions about their role and significance in the environment. Compared to common habitat microbes, microbial culture from barren environments still faces challenges. The nutrient level of desert soil is much lower than that of laboratory medium, and microorganisms living in nutrient-poor conditions for a long time are often unable to withstand the pressure of high-nutrient environments [1]. The total organic carbon content in the desert is generally less than 3.5 g/kg, while the carbon content of the LB medium commonly used in the laboratory is about 7.567 g/L, and the carbon content of the NA medium is about 6.68 g/L. As a result, it has become particularly difficult to obtain oligotrophic microorganisms from extreme environments using traditional isolation and culture techniques [2].

Kuznetsov et al. [3] defined oligotrophic bacteria as those capable of growth on media with a carbon content of 1–15 mg/L. Oligotrophic bacteria play a key role in low-nutrient ecosystems [4]. The nutritional requirements for these microorganisms have increased with the rapid progress of molecular biology technology [1,5], necessitating a deeper understanding of ecological roles in nutrient-poor conditions. In particular, researchers have focused on the isolation and culture, distribution patterns, nutrient acquisition strategies, and functions of nutrient-poor microbial species with ecological conservation value [6,7,8,9]. For example, Hui et al. [10] isolated an oligotrophic bacterium that secretes a large amount of mucopolysaccharides from desert crust in Xinjiang. The crust formed by this bacterium helps improve the stability of surface sandy soil and slow the evaporation of soil water, demonstrating its ecological adaptability and importance in arid environments. These studies not only reveal the diversity and importance of oligotrophic bacteria in the natural environment but also highlight the importance of further research into the role of these microorganisms in different ecosystems for ecological conservation and sustainable development.

To enhance microbial resource availability and investigate microbial diversity, researchers worldwide are striving to refine microbial culture technologies and develop novel culturing methodologies. For instance, Button et al. [11] used highly diluted seawater samples to increase the likelihood of successful cultivation by ensuring that the sample contained only a few or even a single microbial cell. Cho [12] utilized oligotrophic media derived from Pacific coastal separations and oceanic samples, successfully isolating oligotrophic marine Gammaproteobacteria while also revealing greater microbial diversity in dilution media. Zhang et al. [13] collected samples from five distinct arid desert regions with varying landforms in Xinjiang, China, yielding five strains of oligotrophic bacteria; they recommended that organic carbon content in low-nutrient environments should not exceed 50 mg/L during microorganism cultivation. Some researchers emphasize the importance of monitoring nutrient component concentrations when cultivating samples in oligotrophic conditions [14]. Additionally, the duration of cultivation is critical for isolating and cultivating microorganisms. Indeed, Leadbetter et al. [15] examined the correlation between the duration of cultivation and slow-growing microorganisms, discovering that it may take up to 5 weeks for a single cell to form a visible colony. Janssen et al. [16] demonstrated that previously uncultivated microorganisms could be obtained through an extended duration of cultivationstrategy, leading to the discovery of multiple new strains of Acidobacteria and Verrucomicrobia; hence, extending the duration of cultivation is essential for successfully isolating slow-growing microorganisms.

Currently, extreme dilution methods—either applied directly to samples or within growth media—are predominantly used for isolating microbes in oligotrophic environments; however, research on oligotrophic bacteria has primarily focused on aquatic ecosystems, while studies concerning their presence in soil and desert habitats remain scarce. Consequently, the role of oligotrophic bacteria within degraded terrestrial ecological systems remains insufficiently explored. Moreover, no studies have established multiple media with gradient concentration series combined with prolonged culture durations across various samples to assess how medium nutrient levels influence microbial isolation and cultivation. In this study, we report on the isolation of culturable microorganisms from soil samples collected from the Taklimakan Desert using straightforward techniques aimed at providing new perspectives into bacterial diversity present within this environment to explore the influence of the medium’s nutrient level on isolation and culture of bacteria in Taklimakan desert, with a focus on addressing challenges associated with unculturable or difficult-to-culture organisms and enhancing their culturability through synergistic approaches.

We hypothesized that nutrient levels within growth mediums significantly limit microbial proliferation; furthermore, microorganisms might have adapted over time to endure long-term nutrient scarcity typical of low-nutrient settings similar to those found in the Taklimakan Desert. Thus, preparing media characterized by lower nutrient concentrations may facilitate more effective isolation and cultivation strategies for microbes sourced from such impoverished environments. We investigated how variations in medium nutrient levels affect microbial isolation and cultivation processes specific to conditions prevalent in the Taklimakan Desert, while offering new ideas and strategies applicable to similar barren ecosystems.

## 2. Materials and Methods

### 2.1. Test Samples

The Tarim Basin Desert covers an area of 3,376,000 square kilometers, with 28.5% of its surface being occupied by active sand dunes, making it the second largest moving desert and China’s largest desert [17]. The Tarim Basin Desert is very arid, with an annual precipitation of less than 25 mm and a long-term average annual precipitation of 1 mm [18] and a potential evapotranspiration of 1000 mm. Its climate is windy, with high solar radiation (http://data.cma.cn, accessed on 20 July 2022) and temperature fluctuations between −20 and 70 °C.

**Figure 1:**
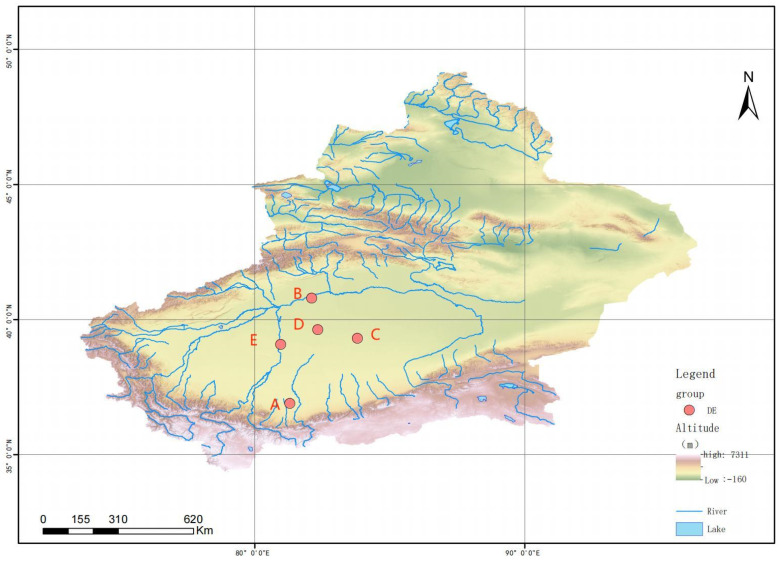
Sample collection points in Taklimakan Desert. Note: A: South, B: North, C: West, D: East, E: Central.

In July 2022, five sampling points were selected in the eastern, western, southern, northern, and central margins of the Tarim Basin Desert, the sampling map is shown in Figure 1, and the sampling information is shown in Table 1. The samples were sealed in Labplas sterile bags (EFR-5590E, TWIRL’EM, Mississauga, ON, Canada) and stored at −20 °C until analyzed.

### 2.2. Measurement of the Physical and Chemical Properties of Soil

The soil pH was measured using a pH meter (PT-10, Sartorius, Gottingen, Germany) with a ratio of fresh soil–water = 1:2.5 (*w*/*v*) [19]. The electrical conductivity (EC) was measured using a conductivity meter (DDSJ-308A, Leici, Shanghai, China) according to the ratio of fresh soil–water = 1:5 (*w*/*v*). The contents of total nitrogen (TN), total carbon (TC), and total organic carbon (TOC) were measured using an elemental analyzer (Elementar Vario-EL, Langenselbold, Hessen, Germany) [20,21,22]. The content of total potassium (TK) was determined using the NaOH melting-flame photometer method; the content of ammonium nitrogen (NH4-N) and nitrate nitrogen (NO3-N) was determined using the KCl leaching-indigo phenol blue colorimetric method. Organic phosphorus (OP) and inorganic phosphorus (IP) were determined using the Moore-Vandal method, while water-soluble organic carbon (DOC) and water-soluble organic nitrogen (DON) were determined by high-temperature catalytic oxidation. For each sample, three parallel measurements were performed for each physicochemical property.

### 2.3. Microbial Isolation and Counting

For microbial isolation and counting, LB [23], R2A [24], and Gao’s No.1 (GS) were used, with a pH of 8.5. Furthermore, 1000 μL of trace salt mother liquor (ferrous sulfate, zinc sulfate, and manganese chloride; 1 g/1000 mL each) was added to 1000 mL medium.

The following four horizontal media were developed according to the oligotrophic bacteria definition:

Eutrophic media: LB and GS medium diluted at 1/2, 1/5, 1/10, and 1/15.

Mestrophic media: LB medium diluted at 1/20 and 1/30 or R2A medium diluted at 1/2 and 1/5.

Oligotrophic media: LB medium diluted at 1/40 and 1/50 or R2A medium diluted at 1/10, 1/15, 1/20, 1/30, 1/40, and 1/50.

Extremely oligotrophic media: LB and R2A media diluted at 1/100, 1/200, and 1/300 and water lipid medium (a medium containing only AGAR and water).

We used a sterile mortar to grind soil samples from the Taklimakan Desert. The specific operation is to wrap sterile cotton in a sterile velvet cloth to form a similar “culture dish” shape, so that it has the same size bottom as the medium; dip into about 0.1 g of soil sample; and replicate it evenly on the surface of the medium plate to separate and count culturable bacteria. For each medium with different dilution concentrations, at least three parallel agar plates were used to meet the minimum requirements for statistical analysis. Samples should be inverted and cultured for more than 30 days at 28 °C in the dark. During the culture period, the growth of microorganisms was observed and recorded, and the effects of different nutrient levels on the diversity and quantity of colonies were analyzed.

Individual colonies grown on the medium were compared based on their morphological characteristics (size: compare the length, width, and height of different bacteria; shape: analyze the outline and symmetry of the sample; color: record the color distribution and pigment concentration of the sample; texture: evaluate the microstructure and roughness of the sample surface). Subsequently, each individual colony was transferred to the corresponding diluted concentration medium for purification. We performed three-zone purification on an AGAR plate using inoculation rings, streaking continuously, and diluting microorganisms through streaking to form individual colonies to obtain pure single-colony bacteria. The collected bacteria were preserved in 20% (*v*/*v*) glycerin and stored at −80 °C for follow-up experiments.

### 2.4. DNA Extraction and 16S Sequencing

The total genomic DNA of bacterial isolates was extracted using a bacterial DNA extraction kit (Omega Bio-Tek, Norcross, GA, USA). The 16S rRNA gene fragment was amplified using the universal primers 27F and 1492R [25]. The PCR reaction cycle conditions were as follows: initial denaturation at 95 °C for 3 min, followed by denaturation at 95 °C for 30 s, annealing at 55 °C for 30 s, elongation at 72 °C for 1 min, and final elongation at 72 °C for 5 min. Sequencing was performed using a PCR instrument (C1000 Touch) sequencer. After agarose gel electrophoresis, samples with clear and bright bands were sent to Shanghai Qingke Biological Co., Ltd. (Shanghai, China) for sequencing. The sequencing results were validated using the EzBioCloud database [26], and the strains were identified. Adjacent connected trees were restored in MEGA 6.0 [27]. Sequences were aligned using ClustalW [28]. The evolutionary distance was calculated using Kimura’s two-parameter model [29]. The confidence of the resulting tree topology was calculated using bootstrapping with 1000 resampling [30].

### 2.5. Data Analysis

DNA extraction was performed using the MP Soil kit (MP Biomedical, Santa Ana, CA, USA, 116564384). The PCR amplification of the V3-V4 variable region of bacterial 16S rRNA was conducted using specific priors 799 F (5′-ACTCCTACGGGAGGCAGCA-3′) and 1193 R (5′-GGACTACHVGGGTWTCTAAT-3′). The desert samples were sequenced using 16s rRNA on the Illumina MiSeq platform (Shanghai Pasenuo Technology Co., Ltd., Shanghai, China). Sequences were processed using Qiime2 (2023.5) software [31] and visualized using R4.3.0 software [32]. The data in the table regarding the physical and chemical properties of the desert samples are the mean ± standard deviation. Different lowercase letters after the same column of data indicate a significant difference at the *p* < 0.05 level tested using Duncan’s new complex range method. The total number of soil microbial plate colonies isolated on media of different nutrient levels using Origin2021’s graph based on mean value and standard deviation.

## 3. Results and Analysis

### 3.1. Physical and Chemical Properties of Sandy Soil in the Taklimakan Desert

The altitudes of the sampling sites varied from 990 to 1364 m above sea level. As presented in Table 2, the conductivity ranges were between 115.2967 and 1986.9233 S/m. The pH levels of samples collected from the Taklimakan Desert exhibited a decrease from 8.72 in the west to 9.8 in the east; the TC, IP, NO3-N, DOC, DON, and EC displayed similar trends. The central, northern, and southern regions showed a decreasing trend; the TC, IP, OP, and TN displayed similar trends. OC, NO3-N, DOC, DON, and EC showed a decreasing trend from south to north (see Table 2).

### 3.2. Species Composition of Different Samples Is Different

**Figure 2:**
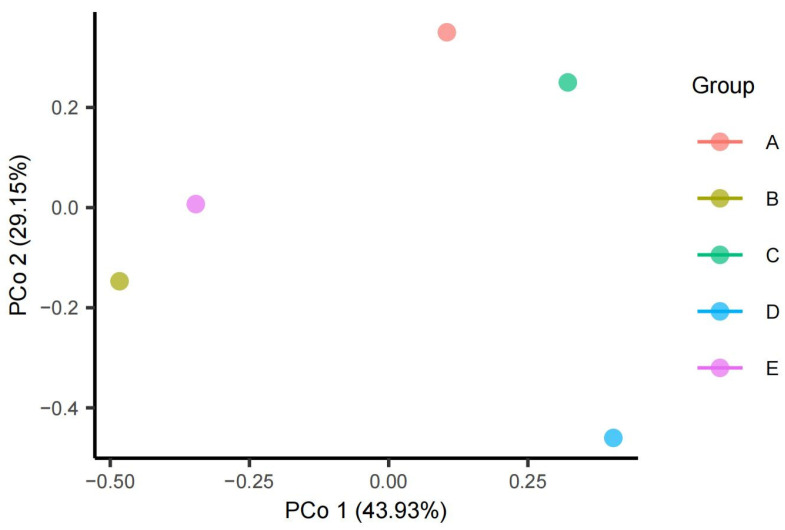
PCoA analysis of five sand samples in the Taklimakan Desert. Note: A: South, B: North, C: West, D: East, E: Central.

Constrained principal coordinate analysis (Constrained PCoA) was used to maximize the difference between samples. PCoA analysis was performed on different sand samples from five directions (east, west, north, south, centel) in the middle of the desert, and the results are shown in Figure 2. Based on the PCoA analysis, the contribution rates of the first principal component (PCo1) and the second principal component (PCo2) were 43.93% and 29.15%, respectively. There was no overlap in the five samples of Taklimakan Desert soil, indicating that the five samples had no obvious classification characteristics, and the five samples had differences in bacterial species. The relative positions of the sample points on the PCoA plot indicate that they are based on Bray–Curtis distances, with sample points D and E relatively close together, while groups A, B, and C are far apart from each other. The results show that the composition and structure of bacteria in the D and E regions were similar, and the differences were small.

### 3.3. High-Throughput Determination of Bacterial Composition in Soil Samples from the Taklimakan Desert

**Figure 3:**
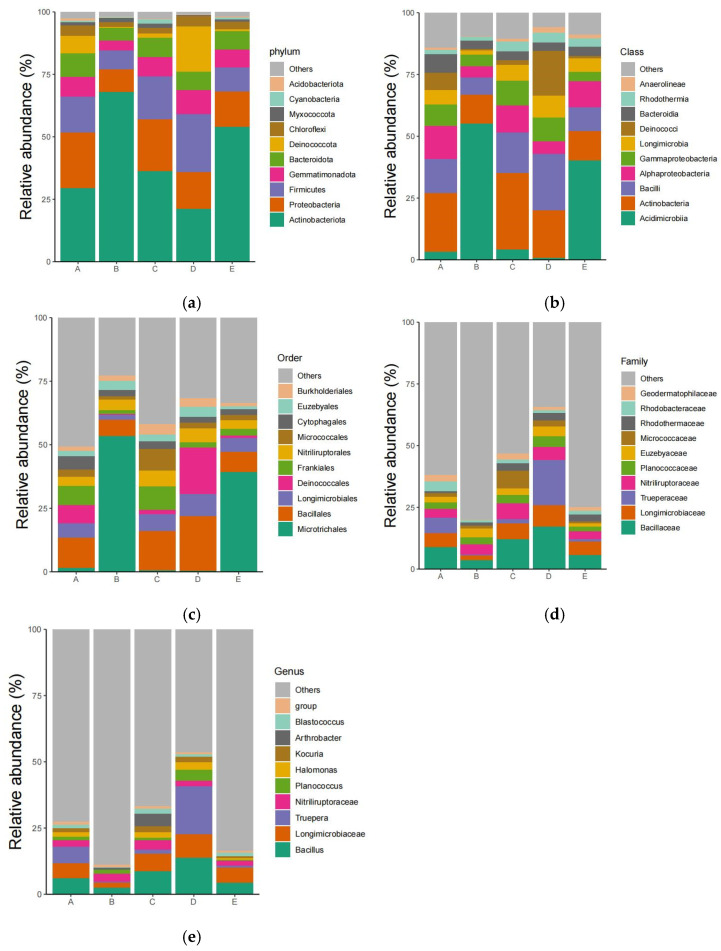
The top ten categories of bacteria at different taxonomic levels: (**a**) phylum, (**b**) class, (**c**) order, (**d**) family, and (**e**) genus. Note: A: South, B: North, C: West, D: East, E: Central.

After the high-throughput bacterial sequencing of the soil samples, 360,500 valid sequences and 2934 unique sequences were obtained. The operable operational taxonomic units (OTUs) were clustered with 97% similarity, and 1908 OTUs were obtained using QIIME2 (2023.5). The Shannon index of microorganisms among samples ranged from 3.302 to 6.348 (Table 3), among which the Shannon index of samples from Yutian County was the highest (6.348), followed by that of samples from Qiemo County (5.745). The Alar City sample had the lowest index. The coverage index was 0.998–0.999, which might reflect the percentage of samples classified at the phylum, class, order, family, and genus levels (Figure 3). *Actinobacteria* accounted for ~30.9% of the total at the phylum level. Other phyla, *Proteobacteria*, *Firmicutes*, *Bacteroidetes*, *Gemmatimonadetes*, and *Chloroflexi*, accounted for >4%. At the class level, *Bacilli* was the most abundant, accounting for 18.6%, while *Actinobacteria*, *Alphaproteobacteria*, *Nitriliruptoria*, *Gemmatimonadetes*, *Cytophagia*, and *Gammaproteobacteria* accounted for >5%. At the order level, *Bacillales* accounted for the largest proportion (18.4%), followed by *Nitriliruptorales*, *Rhizobiales*, and *Frankiales*, accounting for >5%. At the family level, *Bacillaceae* was the most abundant (12.9%), followed by *Nitriliruptoraceae*, *Planococcaceae*, and *Rhodothermaceae*, accounting for >4%. At the genus level, *Bacillus* was the most abundant (9.5%), followed by *Nitriliruptor*, *Truepera*, *Euzebya*, *Pseudomonas*, and *Planococcus*, accounting for >2%. According to the Chinese soil database (http://vdb3.soil.csdb.cn/, accessed on 20 July 2022) from the second national soil census, the “soil fertility classification standard” (GB/T 17296, https://openstd.samr.gov.cn/bzgk/gb/newGbInfo?hcno=D59C90AB5DA4F335F0D2BBFE79893627, accessed on 20 July 2022) recommends dividing the classifications into six phases; the physical and chemical factors of the Taklamakan Desert sand content belong to phase 6, which is the highly oligotrophic environment.

### 3.4. The Number of Colonies Isolated from Different Nutrient Levels of Medium

**Figure 4:**
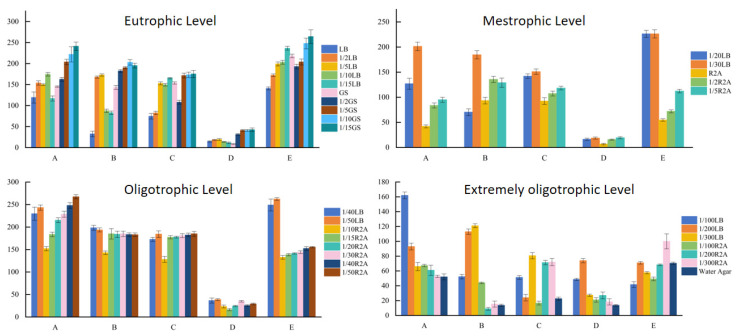
Total number of soil microbial plate colonies isolated on media of different nutrient levels. Note: A: South, B: North, C: West, D: East, E: Central.

Five soil samples collected from areas with different latitudes and longitudes within the Taklimakan Desert were cultured in separate media with different nutrient levels of medium for 20–30 days, and the total number of microbial colonies obtained by plate counting is shown in Figure 4. The overall performance trend indicates that the number of colonies on the plate increases with the decrease in the dilution concentration, and the number of bacteria obtained is higher than the total number of bacteria in the original plate colony. The results show that the low nutrient level medium was more beneficial to the isolation and cultivation of oligotrophic microorganisms from the Taklimakan Desert.

Microorganisms were isolated and cultured under eutrophic level conditions. The total number of colonies obtained by diluting LB and GS medium was higher than that obtained using the original concentration medium, and the soil samples from Yutian County, Qiumang County, Shaya County, and Luopu County had the highest total number of colonies obtained using a 1/15 dilution of the GS medium. Soil samples from Aral City yielded the highest total number of colonies in 1/10 GS diluted medium.

By isolating and culturing microorganisms in nutrient-level culture medium, we revealed that the total number of colonies obtained in LB diluted medium was higher than that in R2A medium and R2A diluted medium, whereas the total number of colonies obtained in R2A diluted medium increased with the decrease in medium concentration. The total number of colonies obtained in R2A diluted medium was higher than that obtained in original R2A medium. The soil samples from Yutian County, Alar City, Qima County, and Shaya County all yielded the highest total number of colonies in 1/30 LB diluted medium, while those from Luopu County yielded the highest total number of colonies in 1/20 LB diluted medium.

By isolating and culturing microorganisms under oligotrophic level culture conditions, we observed that soil samples from Yutian County, Qiemo County, and Luopu County yielded the highest total number of colonies in 1/50 LB diluted medium, whereas soil samples from Alar City yielded the highest total number of colonies in 1/40 LB diluted medium.

When isolating and culturing microorganisms under oligotrophic level conditions, we discovered that the soil sample of Yutian County yielded the maximum number of colonies in 1/100 LB diluted medium. The total number of colonies yielded from soil samples from Alar City was the highest in 1/300 LB diluted medium. The soil samples from Shaya County yielded the largest number of colonies in 1/200 LB diluted medium. The soil samples from Mu County and Luopu County yielded the largest number of colonies in 1/300 R2A diluted medium.

### 3.5. Distribution of Culturable Microbial Species in the Taklimakan Desert

A large number of microbial strains were obtained from samples collected at different points in the Taklimakan Desert through the isolation and culture of diluted medium combined with an extended culture duration. The 16S rRNA-based bacterial species information is shown in Table 4. A total of 669 strains were isolated from the soil of Taklimakan Desert, which were classified into 5 phyla, 7 classes, 25 orders, 42 families, 83 genera, and 379 species. The 16S rRNA gene sequences of 379 non-repeating strains were stored in the GenBank database (entry numbers OR434840-OR435092 and PQ269488-PQ269635). Among them, 148 strains had a maximum similarity lower than 98.65% and were potential new species. Further, 67 new species were obtained from eutrophic-level medium, 21 from Mestrophic-level medium, 23 from oligotrophic-level medium, and 37 from the extremely oligotrophic-level medium.

### 3.6. Microbial Community Structure and Diversity Analysis Based on 16S rRNA Bacterial Species Analysis

#### 3.6.1. Culturable Microbial Community Structure of the Taklimakan Desert

**Figure 5:**
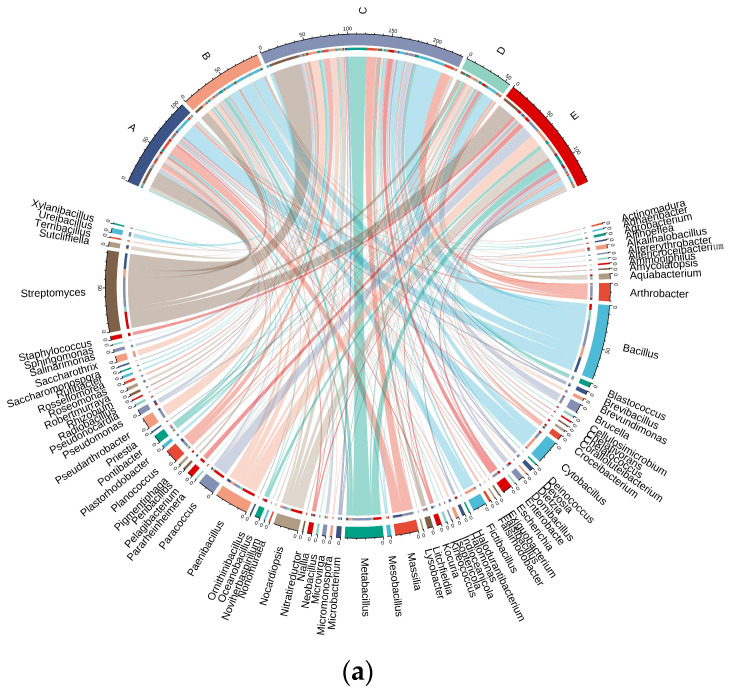

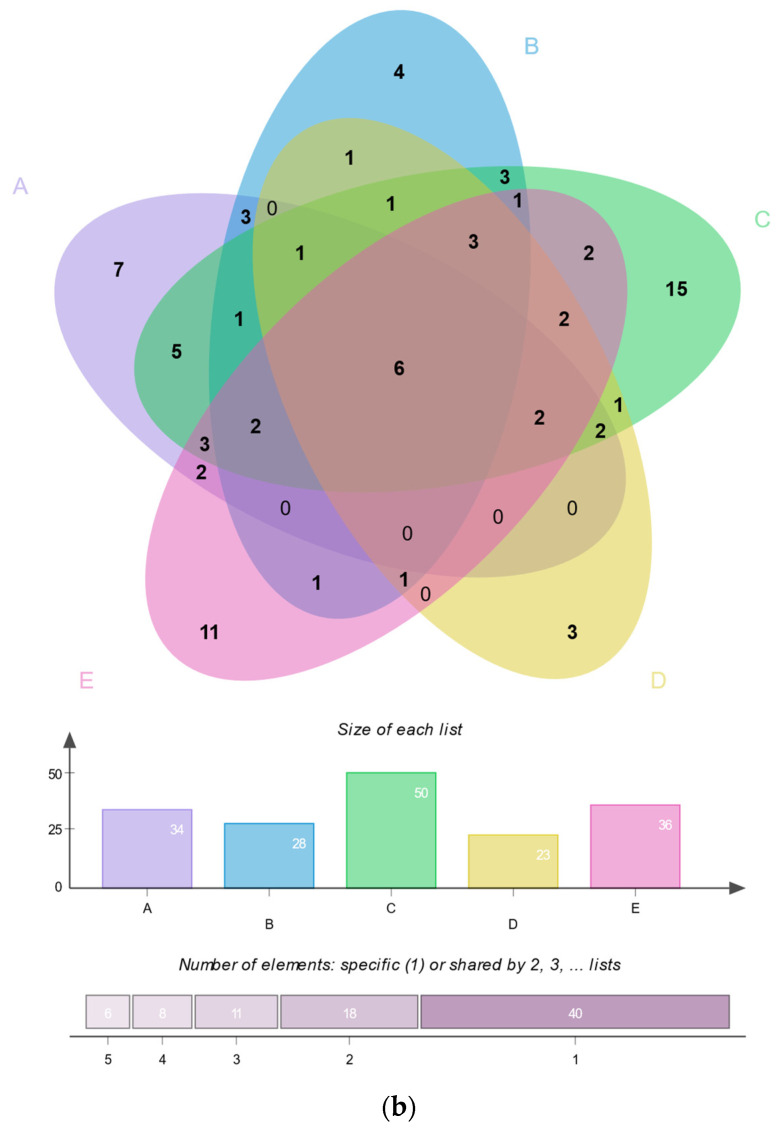
Microorganisms isolated from five soil samples from the Taklimakan Desert. The chord plot (**a**) of the bacterial community in the desert samples represents the difference in the distribution of microorganisms obtained from the five Taklimakan Desert soil samples. The Venn diagram of the bacterial community in the desert sample represents the quantitative distribution of genus (**b**). These samples are represented by different circles: a Venn diagram representing the number of genera detected in each sample and the overlap of genera in the sample.

Culture medium with four nutrient levels was used for the following experiments. The differences in microbial community structure among the samples from the Taklimakan Desert are shown in Figure 5.

A total of 34 genera were isolated from sample A. *Bacillus* and *Streptomyces* were the dominant genera, accounting for 17.92% of the isolates, followed by *Massilias*, accounting for 15.58% of the isolates. Compared with other samples, seven unique bacterial genera were identified from sample A: *Altererythrobacter*, *Altericroceibacterium*, *Enterobacte*, *Isoptericola*, *Peribacillus*, *Rufibacter*, and *Xylanibacillus*.

Twenty-eight genera were isolated from sample B, of which *Cytobacillus* accounted for the highest proportion (16.67%), followed by *Bacillus* (14.58%). In addition, compared with other samples, four unique bacterial genera were identified by sample B isolation: *Exiguobacterium*, *Falsibacillus*, *Ornithinibacillus*, and *Saccharomonospora*.

A total of 50 genera were isolated from sample C, of which *Streptomyces* accounted for the highest proportion (15.45%), followed by *Bacillus* (13.73%). Sample C isolation identified 15 unique bacterial genera compared to other samples: *Actinomadura*, *Adhaeribacter*, *Chelatococcus*, *Dietzia*, *Falsirhodobacter*, *Halodurantibacterium*, *Kineococcus*, *Lysobacter*, *Nonomuraea*, *Noviherbaspirillum*, *Plastorhodobacter*, *Radiobacillus*, *Rhizobium*, *Roseomonas*, and *Salinarimonas*.

Twenty-three genera were isolated from sample D. The dominant genera were *Bacillus* and *Streptomyces*, accounting for 23.21% and 10.71%, respectively. Compared with other samples, sample D isolation identified three unique bacterial genera, including *Ammoniphilus*, *Deinococcus*, and *Sphingomonas*.

A total of 36 genera were detected in sample E. *Streptomyces*, accounting for 15.44%, followed by *Paenibacillus* and *Paracoccus* (11.76% and 10.29%, respectively). Compared with other samples, sample E isolation identified 11 unique bacterial genera: *Aliihoeflea*, *Amycolatopsis*, *Chelativorans*, *Coralloluteibacterium*, *Nitratireductor*, *Pararheinheimera*, *Pelagibacterium*, *Pigmentiphaga*, *Priestia*, *Staphylococcus*, and *Terribacillus*.

Five sets of Venn diagrams (Figure 3b) were established for five samples at the genus level, showing the genera shared by two, three, four, and five samples. All samples were members of six genera. At the genus level, major differences in five samples were revealed, with most genera detected in only one sample.

#### 3.6.2. Community Structure of Microorganisms in the Taklimakan Desert under Different Nutrient Levels of Medium

**Figure 6:**
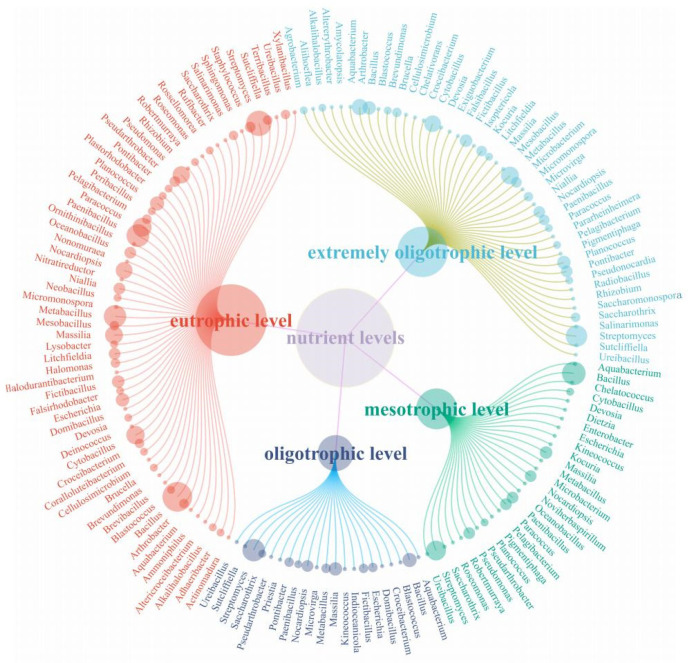
Microorganisms isolated from the Taklimakan Desert at different medium nutrient levels.

Microorganisms from the Taklimakan Desert were isolated and cultured under different medium nutrient levels, and the diversity of microorganisms obtained at the genus level is shown in Figure 6. We discovered 23 genera of microorganisms obtained only at eutrophic levels: *Actinomadura*, *Adhaeribacter*, *Altericroceibacterium*, *Ammoniphilus*, *Brevibacillus*, *Coralloluteibacterium*, *Deinococcus*, *Falsirhodobacter*, *Halodurantibacterium*, *Halomonas*, *Lysobacter*, *Neobacillus*, *Nitratireducto*, *Nonomuraea*, *Ornithinibacillus*, *Peribacillus*, *Plastorhodobacter*, *Rossellomorea*, *Rufibacter*, *Sphingomonas*, *Staphylococcus*, *Terribacillus*, and *Xylanibacillus*. Four bacteria were isolated at the Mestrophic level only, namely *Noviherbaspirillum*, *Chelatococcus*, *Dietzia*, and *Enterobacter*. Two genera were isolated under oligotrophic level culture, namely Indioceanicola and Priestia. Only 10 bacteria genera were isolated under extremely oligotrophic level conditions: *Altererythrobacter*, *Amycolatopsis*, *Chelativorans*, *Exiguobacterium*, *Falsibacillus*, *Isoptericola*, *Pararheinheimera*, *Pseudonocardia*, *Radiobacillus*, and *Saccharomonospora*.

#### 3.6.3. Rare Microorganisms Isolated from Different Medium Nutrient Levels

**Figure 7:**
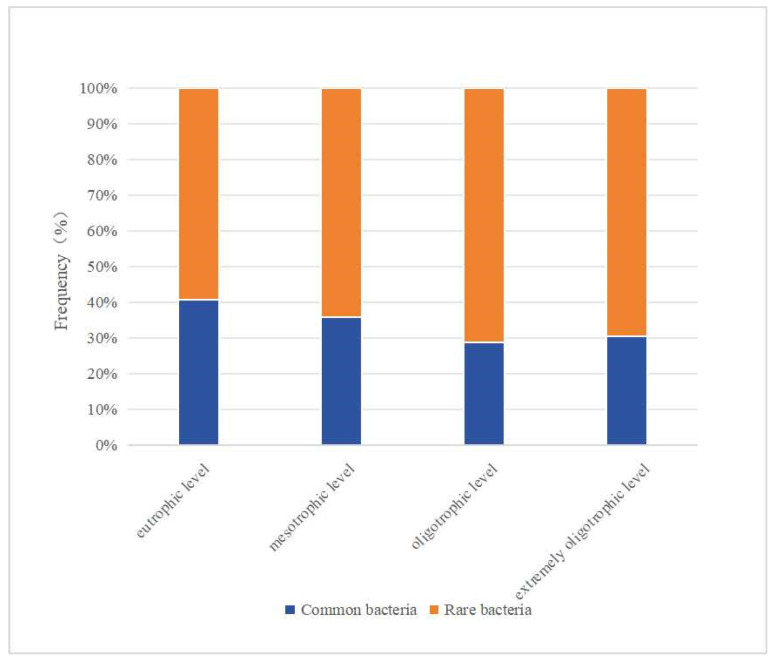
Proportion of rare bacteria in each nutrient level medium.

After soil microbial cultivation under different nutritional conditions, we isolated the yield in 83 genera by comparing their sequence information to that in the List of Prokaryotic names with Standing in Nomenclature database (https://www.bacterio.net/, accessed on 20 July 2022) and determined that most of these species belong to the rare or recently described genera of microbes (Table S1). There are 25 genera with less than 10 published strains, and 59 genera with less than 50 published strains. A total of 69 bacteria genera were obtained at the eutrophication level, and 41 of the published bacteria genera were below 50 published strains, accounting for 68.3%. A total of 28 bacteria genera were obtained at the moderate nutrient level, and 18 bacteria genera (64.3%) were published with a number of bacteria below 50 published strains. A total of 21 bacteria genera were obtained at the oligotrophic level, and 15 of the published bacteria genera were below 50, accounting for 71.4%. A total of 46 bacteria genera were obtained at the extremely oligotrophic level, and 32 of the published bacteria genera were below 50 published strains, accounting for 69.6%. The proportion of rare bacteria in each nutrient level medium is shown in Figure 7. The results show that the reduction in nutrient level in the medium was beneficial for the mining of rare bacterial resources.

### 3.7. Diversity of Culture-Free and Culturable Bacterial Communities in the Taklimakan Desert

The analysis results of culture-free and cultureable methods were compared. The four dominant phyla, including *Proteobacteria*, *Actinobacteria*, *Firmicutes*, and *Bacteroidetes*, and the seventh dominant phyla, *Deinococcus Thermus*, can be cultured and isolated. A total of 123 bacterial genera were detected by high-throughput sequencing, 29 of which were successfully isolated during culturable processes. In the middle of the Taklimakan Desert, 23 of the first 50 detected bacteria genera were successfully isolated. The differences between culture-free and culturable bacteria genera are shown in Figure S1. The comparison between the two methods shows that there are a large number of common taxonomic groups below the taxonomic genus level. This fully indicates that the separation and culture methods combined with velvet cloth replicate, dilution culture, and extended duration of cultivation could effectively isolate more bacteria in the soil of the Taklimakan Desert.

## 4. Discussion

### 4.1. Bacterial Diversity in the Taklimakan Desert

In most desert ecosystems, high-throughput sequencing reveals that *Bacteroidetes*, *Firmicutes*, and *Proteobacteria* are the dominant bacterial groups. The diversity of sand dunes in the Qinghai–Tibet Desert is similar to those in these environments with *Firmicutes*, *Actinobacteria*, *Proteobacteria*, and *Bacteroidetes* as the dominant phyla [33]. Previous studies used pyrosequencing to reveal the composition of microbial communities in surface sandy samples and dust storms in and around the Taklimakan Desert. Four phyla were revealed, including *Proteobacteria*, *Bacteroidetes*, *Actinobacteria*, and *Firmicutes* [34,35]. The dominant phyla in our study are the same as in the studies above, but there are some differences in their abundance levels. This may be because Shu et al. used 16S rDNA V1-V2 variable region amplicons to study the bacterial diversity of surface sand in the Gobi and Taklimakan deserts, whereas we used the V3-V4 region for our study. Secondly, pyrosequencing was used in the above study, while illusmina was used to detect microbial communities. Different amplified fragments and different sequencing methods would lead to deviations in sequencing results [36,37].

Shu et al. suggest that members belonging to four bacterial genera, *Pontibacter*, *Salinimicrobium*, *Planococcus*, and *Marmoricola*, may be classified as indigenous desert types [35]. These four genera of bacteria were also present in our test results. At the same time, Lauber et al.‘s data also contain these several strains [38]. *Planococcus*, a genus belonging to the *Firmicutes* phylum, is known to inhabit alkaline environments such as marine solar salterns and extreme conditions like the cold deserts of the Himalayas and Antarctica. This genus has also been identified in Saharan dust events in Mali, West Africa [39], and in Erdemli, Turkey [40]. Similarly, *Marmoricola*, an *Actinobacteria* genus, has been isolated from diverse settings including volcanic ash [41], marble statues, beaches, and Korean soil [42].

In terms of culturable bacteria, members of *Actinobacteria* are the dominant species [43,44,45,46]. In our study, Gram-positive bacteria (members of *Firmicutes* and *Actinomycetes*) outnumbered Gram-negative bacteria among bacteria cultured using samples from the Taklimakan Desert. In contrast, in a similar ecological environment, the Thar Desert, there were more Gram-negative bacteria (*Proteobacteria* and *Bacteroidetes*) than Gram-positive bacteria (*Firmicutes* and *Actinobacteria*) [47]. However, after pretreatment with 3KGy gamma rays, Yu et al. successfully isolated five phyla, including the above four phyla and *Deinococcus-Thermus* [48], which is consistent with our results. However, we did not use the radiation pretreatment technique, and the number of strains isolated was much smaller than ours.

We selected five soil samples from different latitudes and longitudes in the Taklimakan Desert for parallel comparison and successfully obtained new and uncultured microorganisms from the soil samples using velvet cloth replicate, dilution culture, and extended culture duration. A total of 669 strains were isolated during the 30-day culture cycle, among which 148 strains had a maximum similarity below 98.65% and are potential new species. In addition, among the 2934 new detected sequences, only 1908 OTUs were distinguished, indicating the possible presence of unknown types of bacteria in the Taklimakan Desert. The region is a potential source for the discovery of new bacteria with special characteristics such as alkali and drought tolerance, and a valuable resource for the isolation of novel enzymes [49]. These unique and interesting ecosystems appear to contain numerous unknown bacterial species that still need to be further identified and characterized.

Our results show that *Streptomyces* accounted for 13.19% of the total bacteria and *Bacillus* accounted for 9.76%, which were the dominant strains of culturable microorganisms from the Taklimakan Desert. *Streptomyces* is widely distributed in nature, and its spores and mycelia can be obtained from soil, air, and water, especially in low-water-content, organic-matter-rich, and neutral or slightly alkaline soil. *Streptomyces* shows environmental gradients and regional specificity in some places [50,51,52,53], while *Bacillus* exhibits strong stress resistance in extreme environments and is able to grow at different nutrient levels, with simple nutrient requirements and rapid reproduction characteristics. For example, *Bacillus subtilis* can form endospores and become airborne [54]. These dominant strains reflect the adaptability of microorganisms to extreme environments such as high temperature, drought, and high salinity.

### 4.2. Influence of Nutrient Level on Bacterial Isolation and Culture

Our experimental data reveal the influence of nutrient levels in medium on microbial isolation in the soil of the Taklimakan Desert. The results show that LB, R2A, and GS diluted media were more effective in promoting microbial growth and isolation than traditional eutrophic media. This suggests that an appropriate reduction in nutrient levels can improve the culturability of microorganisms, as certain microorganisms adapted to nutrient-scarce environments may be inhibited under conditions of excess nutrition [55,56]. Traditional microbial culture medium usually has a high nutrient level and relatively simple nutrient elements, while natural environments, especially oligotrophic environments such as the Taklimakan Desert, have much lower medium nutrient levels than laboratory culture conditions. In nutrient-constrained environments, microbes are more likely to cooperate, for example, through metabolite exchange, to improve their chances of survival. In nutrient-rich conditions, competition between species is more intense, which usually favors fast-growing microbes [57].

We also observed that medium with low nutrient levels was more conducive to the isolation of rare microorganisms. This may be because microorganisms that grow slowly and use nutrients efficiently, known as oligotrophs, are more likely to be successfully cultured under nutrient scarcity conditions. These microorganisms act as “marathon runners” in the microbiome, and are able to survive and thrive despite long-term nutritional limitations [57].

It is worth noting that some bacteria can only grow at low nutrient levels; for example, *Microvirga* was isolated in 1/40 LB~1/200 LB in this study, and the growth ability of *Microvirga* on different media has been reported. Species of this genus can be grown in either concentrated media, such as TSA, or diluted media, such as R2A [58], but cannot grow in nutrient-rich environments [59]. Most of the bacterial genera isolated at low nutrient levels belong to less commonly isolated or newly described genera. In particular, 1/300 R2A medium obtained the most unique bacterial genera, indicating that reducing the nutrient level of medium is more conducive to improving the isolation and culture of oligotrophic environmental microorganisms. The results further indicate that the nutrient level of the medium has an important influence on the culturability, diversity, and quantity of microorganisms in extreme environments.

### 4.3. Simple Culture-Based Strategy Can Obtain Numerous Unknown and Rare Bacteria

While we were able to successfully isolate some microorganisms that are difficult to culture with standard methods by diluting the nutrient levels of traditional media, eutrophic media still have their unique value in obtaining unique microbial species. Therefore, the dilution medium method cannot completely replace the traditional culture method. Oligotrophic medium with lower nutrient levels can be used to more effectively screen out bacteria species that are adapted to nutrient-poor environments from the natural environment. Nevertheless, we recognize that some microbial strains may be inherently difficult to culture, which limits the comprehensiveness of monoculture strategies. We adopted the strategy of combining multi-nutrient level medium, which not only supplemented the deficiency of traditional nutrient-rich medium but also provided a more comprehensive method for the study and utilization of soil microbial resources in the Taklimakan Desert, and laid a foundation for subsequent research and application.

In this study, we used sand grinding and velvet cloth replicate methods to isolate and count bacteria. The innovation of these methods is that they can be closer to the natural living state of microorganisms, and the operation is relatively simple and efficient. Sand is a typical porous medium with large pores inside (Figure S2), and its porous nature makes it an adsorption medium for minerals, organic matter, and microorganisms, which may not be fully released using the traditional dilution coating separation method. Therefore, we carried out the fine grinding of desert samples and adopted a velvet cloth replicate method to reduce the adverse effects of moisture on microorganisms in desert environments, thus improving the separation efficiency.

We successfully isolated a variety of difficult-to-cultivate bacteria from the Taklimakan Desert using velvet cloth replicate, dilution culture, and extended culture duration. These findings not only enrich our understanding of desert microbial diversity but also provide a new way to utilize these microbial resources.

## 5. Conclusions

Overall, our results show that reducing nutrient levels in medium is more conducive to isolating more uncultured microorganisms from oligotrophic environmental samples than traditional eutrophic medium. This may not be effective in nutrient-rich soils. Follow-up studies suggest focusing on the concentration and proportion of nutrients in soil and laboratory media. More uncultured bacteria could be isolated from the Taklimakan Desert using the method of velvet cloth replicate, dilution culture, and extended culture duration. This strategy allowed us to retrieve more bacteria with low-16S-rRNA-sequence similarity to any of the described isolates, as well as rare microbes from infrequently isolated or recently described genera. Our research helps to narrow the gap between the genetic diversity of bacteria found in independently cultured next-generation sequencing studies and the ratio of culturable microorganisms. Combining the traditional nutrient-rich medium and the diluted medium with lower nutrient levels not only reflects the diversity of microbial resources more comprehensively but also lays a solid foundation for subsequent research and applications.

## Figures and Tables

**Table 1:** Sample collection information.

No.	A	B	C	D	E
Direction	South	North	West	East	Central
Sampling area	Yutian County County	Alar City	Qiemo County	Shaya County	Lop County
Sample types	Sandy	Sandy	Sandy	Sandy	Sandy
Longitude	81°17′40″ E	82°5′59″ E	83°48′0″ E	82°19′41″ E	80°57′25″ E
Latitude	36°54′9″ N	40°48′0″ N	39°18′50″ N	39°37′52″ N	39°4′59″ N
Altitude (m)	1364	926	990	988	1128.5

**Table 2:** Physicochemical properties of the desert samples.

No.	A	B	C	D	E
TC (g/kg)	(0.5033 ± 0.0120) c	(0.5465 ± 0.0014) a	(0.5292 ± 0.0040) b	(0.3596 ± 0.0042) d	(0.5341 ± 0.0092) ab
IP (g/kg)	(0.4805 ± 0.4810) b	(0.5155 ± 0.5125) a	(0.4828 ± 0.4842) b	(0.3334 ± 0.3341) c	(0.4923 ± 0.4848) b
OP (g/kg)	(0.0168 ± 0.0036) c	(0.0340 ± 0.0026) b	(0.0459 ± 0.0043) a	(0.0287 ± 0.0018) b	(0.0448 ± 0.0053) a
TN (g/kg)	(0.2611 ± 0.0064) b	(0.1272 ± 0.0041) d	(0.1431 ± 0.0011) c	(0.0758 ± 0.0013) e	(0.2754 ± 0.0096) a
TOC (g/kg)	(2.9835 ± 0.1152) a	(1.3887 ± 0.0921) c	(1.7306 ± 0.0447) b	(0.9425 ± 0.0298) d	(2.8418 ± 0.0900) a
NH_4_-N (mg/kg)	(1.0906 ± 0.0505) b	(0.8377 ± 0.1107) c	(1.4432 ± 0.1326) a	(1.5658 ± 0.0611) a	(1.6100 ± 0.14730) a
NO_3_-N (mg/kg)	(60.9560 ± 1.5623) a	(19.8417 ± 0.4272) c	(36.2060 ± 0.2706) b	(7.5463 ± 0.1370) d	(6.1933 ± 0.2146) e
TK (g/kg)	(15.8620 ± 0.0856) c	(15.6539 ± 0.0754) c	(18.4685 ± 0.2643) a	(16.6382 ± 0.2175) b	(16.3900 ± 0.1528) b
DOC (mg/kg)	(54.4943 ± 3.0400) a	(26.2365 ± 11.9005) b	(22.5329 ± 0.2920) b	(7.4250 ± 0.3521) c	(18.6680 ± 1.3207) b
DON (mg/kg)	(7.7808 ± 0.7826) a	(3.9640 ± 0.2978) b	(1.9260 ± 0.2446) c	(1.4253 ± 0.0494) c	(3.6013 ± 0.3176) b
pH	(8.9367 ± 0.0651) c	(9.800 ± 0.1127) a	(9.0600 ± 0.0520) bc	(8.7200 ± 0.0624) b	(9.0933 ± 0.0503) d
EC (S/m)	(1986.9233 ± 3.8618) a	(532.3300 ± 8.5440) c	(1351.6667 ± 7.0946) b	(115.2967 ± 2.5891) e	(169.233 ± 1.1930) d
ASL (m)	1364	1128.5	926	990	998

Note: The data in the table are mean ± standard deviation. Different lowercase letters after the same column of data indicate a significant difference at the *p* < 0.05 level tested using Duncan’s new complex range method. TC: total carbon, IP: inorganic phosphorus, OP: organic phosphorus, TN: total nitrogen, TOC: total organic carbon, NH4-N: ammoniated nitrogen, NO3-N: nitrated nitrogen, TK: total potassium, DOC: dissolved organic carbon, DON: dissolved organic nitrogen, EC: electrolytic, ASL: altitude.

**Table 3:** High-throughput sequencing and α diversity of bacterial communities.

Sample	Observed	Chao1	se.chao1	ACE	se.ACE	Shannon	Simpson	InvSimpson	Fisher	Coverage
A	1489	1504.068	5.952	1503.83	19.21	6.348	0.995	188.001	306.763	0.998
B	772	774.727	2.209	775.92	13.786	3.302	0.717	3.528	125.895	0.999
C	975	975.7	1.022	976.381	14.344	5.745	0.992	120.852	165.762	0.999
D	326	326.231	0.588	327.103	6.366	4.559	0.971	33.899	44.860	0.999
E	1088	1103.294	6.617	1099.633	16.391	4.302	0.845	6.471	188.359	0.999

**Table 4:** Microorganisms isolated and cultured from Taklimakan Desert.

Phylum	Class	Order	Family	Genus	Species
Firmicutes	Bacilli	Bacillales	*Bacillaceae*	*Alkalihalobacillus*	4
				*Bacillus*	37
				*Peribacillus*	1
				*Cytobacillus*	19
				*Domibacillus*	3
				*Fictibacillus*	9
				*Mesobacillus*	8
				*Niallia*	2
				*Neobacillus*	2
				*Oceanobacillus*	2
				*Ornithinibacillus*	1
				*Priestia*	1
				*Robertmurraya*	2
				*Rossellomorea*	1
				*Sutcliffiella*	4
				*Litchfieldia*	5
				*Metabacillus*	22
				*Terribacillus*	1
				*Radiobacillus*	1
				*Falsibacillus*	1
			*Paenibacillaceae*	*Paenibacillus*	12
				*Brevibacillus*	2
				*Xylanibacillus*	1
				*Ammoniphilus*	1
			*Exiguobacteriaceae*	*Exiguobacterium*	1
			*Staphylococcaceae*	*Staphylococcus*	1
			*Planococcaceae*	*Ureibacillus*	4
				*Planococcus*	11
Bacteroidetes	Cytophagia	Cytophagales	*Hymenobacteraceae*	*Adhaeribacter*	2
				*Pontibacter*	9
				*Rufibacter*	1
Proteobacteria	Alphaproteobacteria	Sphingomonadales	*Sphingomonadaceae*	*Sphingomonas*	1
			*Erythrobacteraceae*	*Altericroceibacterium*	1
				*Altererythrobacter*	1
				*Croceibacterium*	5
		Rhizobiales	*Brucellaceae*	*Brucella*	5
			*Chelatococcaceae*	*Chelatococcus*	2
			*Devosiaceae*	*Devosia*	4
			*Methylobacteriaceae*	*Microvirga*	4
			*Phyllobacteriaceae*	*Nitratireductor*	1
				*Chelativorans*	1
				*Aliihoeflea*	1
			*Devosiaceae*	*Pelagibacterium*	3
			*Rhizobiaceae*	*Rhizobium*	2
				*Agrobacterium*	2
			*Salinarimonadaceae*	*Salinarimonas*	2
		Rhodobacterales	*Rhodobacteraceae*	*Paracoccus*	7
				*Plastorhodobacter*	4
				*Falsirhodobacter*	2
				*Halodurantibacterium*	1
		Rhodospirillales	*Azospirillaceae*	*Indioceanicola*	1
			*Acetobacteraceae*	*Roseomonas*	2
		Caulobacterales	*Caulobacteraceae*	*Brevundimonas*	3
	Betaproteobacteria	Burkholderiales	*Comamonadaceae*	*Aquabacterium*	4
			*Oxalobacteraceae*	*Massilia*	11
				*Noviherbaspirillum*	1
Actinobacteria	Gammaproteobacteria	Lysobacterales	*Alcaligenaceae*	*Pigmentiphaga*	2
			*Lysobacteraceae*	*Coralloluteibacterium*	2
		Alteromonadales	*Alteromonadceae*	*Pararheinheimera*	1
	Actinomycetia	Oceanospirillales	*Halomonadaceae*	*Lysobacter*	1
				*Halomonas*	2
		Pseudomonadales	*Pseudomonadaceae*	*Pseudomonas*	4
		Enterobacterales	*Enterobacteriaceae*	*Enterobacter*	1
		Micrococcales	*Micrococcaceae*	*Escherichia*	5
				*Arthrobacter*	15
		Geodermatophilales	*Geodermatophilaceae*	*Blastococcus*	4
		Mycobacteriales	*Dietziaceae*	*Dietzia*	1
		Kineosporiales	*Kineosporiaceae*	*Kineococcus*	2
		Micromonosporales	*Micromonosporaceae*	*Micromonospora*	2
		Micrococcales	*Micrococcaceae*	*Kocuria*	5
		Streptosporangiales	*Microbacteriaceae*	*Microbacterium*	5
			*Nocardiopsaceae*	*Nocardiopsis*	16
		Micrococcales	*Micrococcaceae*	*Pseudarthrobacter*	4
		Pseudonocardiales	*Pseudonocardiaceae*	*Saccharothrix*	5
				*Pseudonocardia*	2
				*Saccharomonospora*	1
				*Amycolatopsis*	1
		Streptomycetales	*Streptomycetaceae*	*Streptomyces*	50
		Cellulomonadales	*Thermomonosporaceae*	*Nonomuraea*	1
				*Actinomadura*	1
			*Promicromonosporaceae*	*Cellulosimicrobium*	2
				*Isoptericola*	1
Deinococcus-Thermus	Deinococci	Deinococcales	*Deinococcaceae*	*Deinococcus*	1

## Data Availability

The datasets generated for this study can be found in GenBank under accession numbers OR434840-OR435092 and PQ269488-PQ269635.

## Supplementary Materials

The following supporting information can be downloaded at https://www.mdpi.com/article/10.3390/biology13100797/s1: Figure S1: Diversity difference between culture-free and culturable strains; Figure S2: Electron microscope image of sand grains; Table S1: The number of existing published strains in LPSN for each nutrient level isolated from the genus obtained.
