# Supplementary material for: Microbial Community Structure in the Taklimakan Desert: The Importance of Nutrient Levels in Medium and Culture Methods
Source: Biology (Basel). 2024 Oct 6;13(10):797. doi: 10.3390/biology13100797 (PMC11505249; doi:10.3390/biology13100797)
Supplement: Supplementary file 1 [file biology-13-00797-s001.zip › biology-3236964-supplementary.pdf]

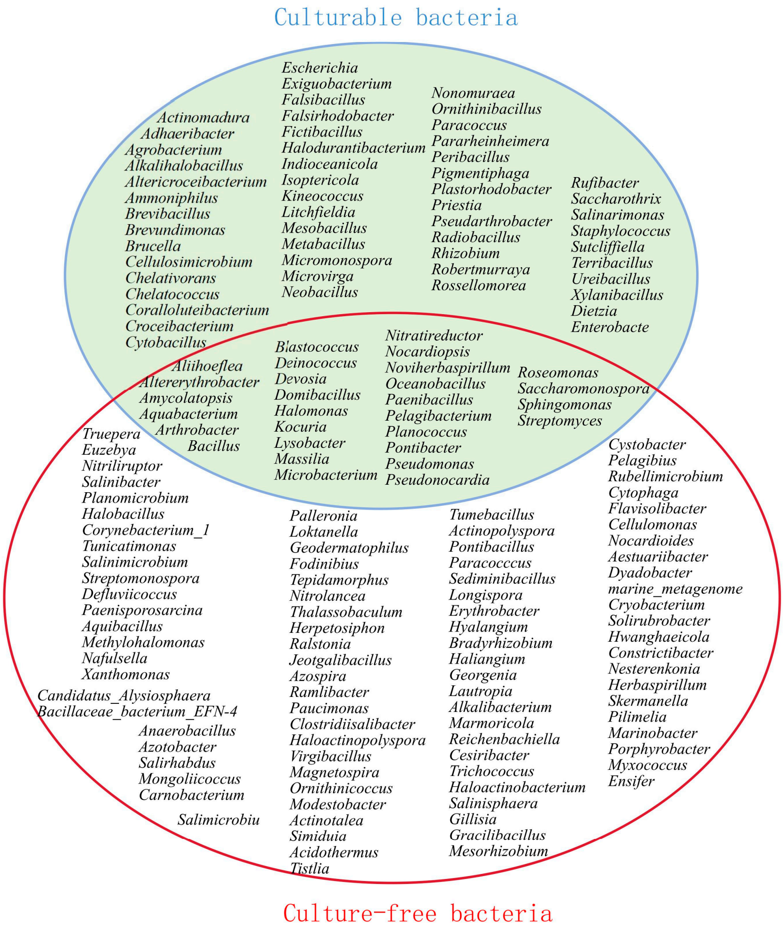

Figure S1. Diversity difference between culture-free and culturable strains.

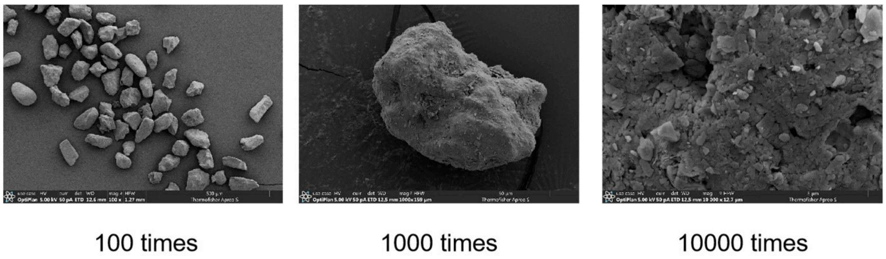

Figure S2. Electron microscope image of sand grains.

Table S1: The number of existing published strains in LPSN for each nutrient level isolated from the genus obtained.

Nutrient levels	Genus	Quantity obtained by separation	LPSN Number of published files
eutrophic level	*Actinomadura*	1	124
eutrophic level	*Adhaeribacter*	2	12
eutrophic level	*Alkalihalobacillus*	2	45
eutrophic level	*Altericroceibacterium*	1	4
eutrophic level	*Ammoniphilus*	1	3
eutrophic level	*Aquabacterium*	1	12
eutrophic level	*Arthrobacter*	3	171

eutrophic level	*Bacillus*	46	2736
eutrophic level	*Blastococcus*	2	17
eutrophic level	*Brevibacillus*	1	35
eutrophic level	*Brevundimonas*	1	41
eutrophic level	*Brucella*	2	34
eutrophic level	*Cellulosimicrobium*	1	10
eutrophic level	*Coralloluteibacterium*	2	2
eutrophic level	*Croceibacterium*	3	12
eutrophic level	*Cytobacillus*	15	20
eutrophic level	*Deinococcus*	1	104
eutrophic level	*Devosia*	2	46
eutrophic level	*Domibacillus*	1	9
eutrophic level	*Escherichia*	7	14
eutrophic level	*Falsirhodobacter*	2	3
eutrophic level	*Fictibacillus*	6	14
eutrophic level	*Halodurantibacterium*	1	1
eutrophic level	*Halomonas*	3	182
eutrophic level	*Litchfieldia*	5	4
eutrophic level	*Lysobacter*	2	97
eutrophic level	*Massilia*	14	91
eutrophic level	*Mesobacillus*	3	16
eutrophic level	*Metabacillus*	24	23
eutrophic level	*Micromonospora*	1	182
eutrophic level	*Neobacillus*	2	27
eutrophic level	*Niallia*	5	6
eutrophic level	*Nocardiopsis*	12	72
eutrophic level	*Nonomuraea*	1	76
eutrophic level	*Oceanobacillus*	5	42
eutrophic level	*Ornithinibacillus*	1	13
eutrophic level	*Paenibacillus*	25	400
eutrophic level	*Paracoccus*	16	108
eutrophic level	*Pelagibacterium*	3	10
eutrophic level	*Peribacillus*	2	22
eutrophic level	*Planococcus*	8	37
eutrophic level	*Plastorhodobacter*	2	1
eutrophic level	*Pontibacter*	2	57
eutrophic level	*Pseudarthrobacter*	4	14
eutrophic level	*Pseudomonas*	13	664
eutrophic level	*Rhizobium*	1	425
eutrophic level	*Robertmurraya*	2	9
eutrophic level	*Roseomonas*	1	115
eutrophic level	*Rossellomorea*	2	6

eutrophic level	*Rufibacter*	1	13
eutrophic level	*Saccharothrix*	1	39
eutrophic level	*Salinarimonas*	3	3
eutrophic level	*Sphingomonas*	1	223
eutrophic level	*Staphylococcus*	4	89
eutrophic level	*Streptomyces*	32	1217
eutrophic level	*Sutcliffiella*	3	8
eutrophic level	*Terribacillus*	1	5
eutrophic level	*Ureibacillus*	3	18
eutrophic level	*Xylanibacillus*	1	1
eutrophic level	*Nitratireductor*	1	18
mesotrophic level	*Aquabacterium*	1	12
mesotrophic level	*Bacillus*	27	2736
mesotrophic level	*Chelatococcus*	1	10
mesotrophic level	*Cytobacillus*	2	20
mesotrophic level	*Devosia*	1	46
mesotrophic level	*Dietzia*	1	21
mesotrophic level	*Enterobacter*	1	59
mesotrophic level	*Escherichia*	1	14
mesotrophic level	*Kineococcus*	1	24
mesotrophic level	*Kocuria*	1	35
mesotrophic level	*Massilia*	6	91
mesotrophic level	*Metabacillus*	1	23
mesotrophic level	*Microbacterium*	1	178
mesotrophic level	*Nocardiopsis*	5	72
mesotrophic level	*Noviherbaspirillum*	1	17
mesotrophic level	*Oceanobacillus*	1	42
mesotrophic level	*Paenibacillus*	5	400
mesotrophic level	*Paracoccus*	5	108
mesotrophic level	*Pelagibacterium*	1	10
mesotrophic level	*Pigmentiphaga*	1	6
mesotrophic level	*Planococcus*	2	37
mesotrophic level	*Pseudarthrobacter*	1	14
mesotrophic level	*Pseudomonas*	5	664
mesotrophic level	*Robertmurraya*	1	9
mesotrophic level	*Roseomonas*	1	115
mesotrophic level	*Saccharothrix*	1	39
mesotrophic level	*Streptomyces*	18	1217
mesotrophic level	*Ureibacillus*	1	18
oligotrophic level	*Aquabacterium*	1	12
oligotrophic level	*Bacillus*	8	2736
oligotrophic level	*Blastococcus*	1	17

oligotrophic level	*Croceibacterium*	1	12
oligotrophic level	*Domibacillus*	1	9
oligotrophic level	*Escherichia*	3	14
oligotrophic level	*Fictibacillus*	3	14
oligotrophic level	*Indioceanicola*	1	1
oligotrophic level	*Kineococcus*	1	24
oligotrophic level	*Massilia*	7	91
oligotrophic level	*Metabacillus*	3	23
oligotrophic level	*Microvirga*	1	37
oligotrophic level	*Nocardiopsis*	5	72
oligotrophic level	*Paenibacillus*	2	400
oligotrophic level	*Pontibacter*	3	57
oligotrophic level	*Priestia*	1	11
oligotrophic level	*Pseudarthrobacter*	1	14
oligotrophic level	*Saccharothrix*	2	39
oligotrophic level	*Streptomyces*	24	1217
oligotrophic level	*Sutcliffiella*	1	8
oligotrophic level	*Ureibacillus*	1	18
extremely oligotrophic level	*Agrobacterium*	2	33
extremely oligotrophic level	*Aliihoeflea*	1	1
extremely oligotrophic level	*Alkalihalobacillus*	2	45
extremely oligotrophic level	*Altererythrobacter*	1	51
extremely oligotrophic level	*Amycolatopsis*	1	101
extremely oligotrophic level	*Aquabacterium*	1	12
extremely oligotrophic level	*Arthrobacter*	11	171
extremely oligotrophic level	*Bacillus*	7	2736
extremely oligotrophic level	*Blastococcus*	1	17
extremely oligotrophic level	*Brevundimonas*	2	41
extremely oligotrophic level	*Brucella*	3	34
extremely oligotrophic level	*Cellulosimicrobium*	1	10
extremely oligotrophic level	*Chelativorans*	1	7
extremely oligotrophic level	*Croceibacterium*	1	12
extremely oligotrophic level	*Cytobacillus*	12	20
extremely oligotrophic level	*Devosia*	1	46
extremely oligotrophic level	*Exiguobacterium*	1	24
extremely oligotrophic level	*Falsibacillus*	1	2
extremely oligotrophic level	*Fictibacillus*	6	14
extremely oligotrophic level	*Isoptericola*	1	13
extremely oligotrophic level	*Kocuria*	4	35
extremely oligotrophic level	*Litchfieldia*	2	4
extremely oligotrophic level	*Massilia*	1	91
extremely oligotrophic level	*Mesobacillus*	1	16

extremely oligotrophic level	*Metabacillus*	14	23
extremely oligotrophic level	*Microbacterium*	4	178
extremely oligotrophic level	*Micromonospora*	1	182
extremely oligotrophic level	*Microvirga*	3	37
extremely oligotrophic level	*Niallia*	1	6
extremely oligotrophic level	*Nocardiopsis*	5	72
extremely oligotrophic level	*Paenibacillus*	4	400
extremely oligotrophic level	*Paracoccus*	2	108
extremely oligotrophic level	*Pararheinheimera*	1	8
extremely oligotrophic level	*Pelagibacterium*	2	10
extremely oligotrophic level	*Pigmentiphaga*	1	6
extremely oligotrophic level	*Planococcus*	7	37
extremely oligotrophic level	*Pontibacter*	5	57
extremely oligotrophic level	*Pseudonocardia*	2	77
extremely oligotrophic level	*Radiobacillus*	1	2
extremely oligotrophic level	*Rhizobium*	1	425
extremely oligotrophic level	*Saccharomonospora*	1	21
extremely oligotrophic level	*Saccharothrix*	3	39
extremely oligotrophic level	*Salinarimonas*	1	3
extremely oligotrophic level	*Streptomyces*	22	1217
extremely oligotrophic level	*Sutcliffiella*	1	8
extremely oligotrophic level	*Ureibacillus*	1	18
